# Knowledge, practice, and infection status of prisoners toward dermatomycosis: a study in Nepal

**DOI:** 10.3389/fpubh.2024.1406346

**Published:** 2024-07-02

**Authors:** Bikrant Dhakal, Bhagat Lal Shrestha, Sangam Shah, Shrija Rijal, Bibek Bahadur Karki, Paras Modi Pangeni, Nikita Bhatta, Rachana Mehta, Neeraj Thapa, Bushra Zeeshan, Ranjit Sah, Camila Luna, D. Katterine Bonilla-Aldana, Alfonso J. Rodriguez-Morales

**Affiliations:** ^1^Central Jail Hospital, Kathmandu, Nepal; ^2^Nepal Skin & Aesthetic Centre, Kathmandu, Nepal; ^3^Institute of Medicine, Tribhuvan University, Kathmandu, Nepal; ^4^Institute of Medicine, Central Department of Public Health, Kathmandu, Nepal; ^5^Medical Laboratories Techniques Department, AL-Mustaqbal University, Hillah, Babil, Iraq; ^6^Dr Lal PathLabs Nepal, Kathmandu, Nepal; ^7^Nepal Medical College and Teaching Hospital, Kathmandu, Nepal; ^8^Harvard Medical School, Boston, MA, United States; ^9^Green City Hospital, Kathmandu, Nepal; ^10^Department of Microbiology, Dr. D. Y. Patil Medical College, Hospital and Research Centre, Dr. D. Y. Patil Vidyapeeth, Pune, Maharashtra, India; ^11^Department of Public Health Dentistry, Dr. D.Y. Patil Dental College and Hospital, Dr. D.Y. Patil Vidyapeeth, Maharashtra, India; ^12^Faculty of Health Sciences, Universidad Científica del Sur, Lima, Peru; ^13^Research Unit, Universidad Continental, Huancayo, Peru; ^14^Gilbert and Rose-Marie Chagoury School of Medicine, Lebanese American University, Beirut, Lebanon; ^15^Grupo de Investigación Biomedicina, Faculty of Medicine, Fundación Universitaria Autónoma de las Américas-Institución Universitaria Visión de las Américas, Pereira, Colombia

**Keywords:** dermatophytosis, tinea, prisoners, knowledge, hygiene practice, Nepal

## Abstract

**Background:**

Dermatophytosis, commonly known as tinea, poses a significant public health concern worldwide, especially in environments with poor hygiene and overcrowding, such as prisons. Despite its prevalence and impact on quality of life, there is a lack of research on the knowledge and hygiene practices regarding dermatophytosis among prisoners, particularly in Nepal.

**Objective:**

The study aimed to assess prisoners’ knowledge, hygiene practice and infection status regarding dermatophytosis in Central Prison, Nepal.

**Methods:**

A descriptive cross-sectional study with a sample size of 184 respondents was designed to collect data using a validated pre-tested questionnaire from September 2023 to January 2024. The collected data was then analyzed using IBM SPSS version 21. Knowledge and hygiene practices were measured on an eight and 11-point scale and rated as poor (≤4) and sound (>4), bad (≤6), and good (>6), respectively. Summary data were presented by descriptive, while Chi-square and logistic regression were used for inferential statistics at *p* < 0.05.

**Results:**

The findings revealed moderate knowledge among prisoners regarding dermatophytosis, with significant gaps in understanding its spread and prevention. While most prisoners recognized the importance of treatment, there were misconceptions about the inevitability of contracting dermatophytosis and the role of personal hygiene.

**Conclusion:**

Despite good knowledge levels, adherence to recommended hygiene practices was suboptimal, highlighting the need for targeted interventions. The study underscores the importance of addressing knowledge gaps, changing attitudes, and promoting hygienic practices to mitigate the burden of dermatophytosis among prisoners.

## Introduction

Dermatophytosis, also called “tinea,” is a contagious disease caused by a fungus called dermatophytes. Dermatophytes cause superficial and self-limiting infections that can be disfiguring and uncomfortable. Contact with spores, along with skin injury, high temperature, and humidity, increases the susceptibility to infection (1). Most clinical manifestations include blistering, fissures, scales, or spots (2). As dermatophytes thrive in warm and moist environments, the burden of some fungal diseases is more significant in regions with crowded living conditions and poor hygiene (3). Studies have uncovered several themes, including a lack of knowledge of the disease, comprehension of its risk factors, prevention, the significance of traditional and personal beliefs, and care-seeking behavior regarding dermatophytosis (4).

Prisons in developing countries have poor living conditions, lack of personal hygiene, sharing of personal items, and overcrowding, due to which skin diseases are pretty common among inmates (5, 6). In the prisons of Nepal, there is overcrowding, lack of primary care, and inadequate health care (7). A study regarding the pattern of skin diseases in prisoners has been done in Nepal, and it shows that skin infections are the most common dermatological problem (6). In the same study, the prevalence of skin problems was 27.23%. There are no skin infection prevention programs targeted toward prisoners in Nepal. In a similar study in India, despite the participants demonstrating good knowledge about the mode of spread of dermatophytosis, most participants still failed to implement the knowledge in maintaining personal hygiene practices (8).

Dermatophytosis patients experience psychosocial stress and challenges. A study showed significant impairment of quality of life in dermatophytosis patients, where most reported symptoms of itching, embarrassment, and self-consciousness (9). According to a study in India, there is an urgent need to dispel myths or conventional views not backed by facts. The attitude of patients toward dermatophytosis plays a significant role in their willingness to seek medical help and adhere to prescribed treatments (4). The study assesses prisoners’ knowledge and hygiene practices toward dermatophytosis in Central Prison, Nepal.

## Methods

Using structured questionnaires, a descriptive cross-sectional study was carried out between September 2023 and January 2024 among 184 Central Prison, Nepal prisoners. Simple random sampling was used to select the participants, and the minimum required sample size was determined using Cochran’s formula:

$n={Z^{2}}^{*}\;p^{*}q/e^{2}$

Where, *n* = sample size.

*Z* = Level of confidence, at *α* = 0.05, *Z* = 1.96 for 95% CI.

*p* = prevalence of dermatophytosis from a previous similar study, 7.78% (5).

q=1−p=1−0.078=0.922

*e* = margin of error, 5%.

Substituting, *n* = 111.

Taking a 10% non-response rate, the final sample size was 122.

### Sampling

The study purposely selected Central Prison, Nepal. The list of prisoners was obtained from the administrative record, and the required number of participants was obtained using a random number table.

### Inclusion and exclusion criteria

The study focused on a vulnerable and high-risk population, with participation limited to prisoners who provided written consent. Research assistants also engaged with prison inmates in their dormitories to ensure respondent confidentiality. However, the study did not include individuals who were severely ill or on assignment outside the prison during the survey period.

### Data collection and study variables

The pre-tested standardized questionnaire used in this study consisted of three sections, filled out by the interviewer by interviewing participants:

Section A: Socio-demographic characteristics such as age, gender, level of education, marital status were included.

Section B: This section included eight items on source of information, knowledge on transmission, signs and symptoms, and prevention of dermatophyte infection. Eight questions had “Yes” or “No” answers. Knowledge scores were further categorized into two categories: good knowledge and poor knowledge.

Section C: An 11-item question on general hygiene practice focusing on the general hand-washing and showering practices, undergarments drying, nail trimming, frequency of washing towels and other practices such as sharing hair-brush and clothes. They were evaluated based on responses “Yes” (given as one mark) and “No” (given as zero marks). A cut-off point using its mean value was used to categorize the scores into excellent and poor hygiene practices.

Infection Status: It was assessed based on previous diagnosis. The diagnosis was done by the dermatologist based on the history, clinical examination, and relevant laboratory investigations.

### Data analysis

Analysis was conducted using IBM Statistical Package for Social Sciences (SPSS) version 21.0 for Windows. Continuous variables were summarized using means and standard deviations, while categorical data were presented as proportions and percentages in summary tables and charts. The relationship between socio-demographic characteristics and relevant variables was assessed using Chi-square (*χ*^2^) and logistic regression statistics. Respondents’ knowledge of dermatophytosis was assessed on an 8-point scale, categorized as poor (≤4) or good (>4). Similarly, hygiene practices were scored on an 11-point scale and categorized as poor (≤6) or good (>6). At the same time, infection status was assessed by asking about the previous diagnosis. A *p*-value of less than 0.05 was considered statistically significant.

## Results

### Sociodemographic characteristics

The mean age of respondents was 35.70 years, ranging between 19 and 77. The percentage of male respondents was 70.1%, whereas 29.9% were female. In addition, more than ⅔ of respondents (70.1%) were married, and 61.5% had education equivalent to secondary level and above (Tables 1–5).

**Table 1:** Socio-demographic characteristics of the study population.

Characteristics		*N* (%)
Age category	<20 Years	6 (3.3)
	20–40 Years	126 (68.5)
	40–60 years	43 (23.4)
	>60 Years	9 (4.9)
Gender	Male	129 (70.1)
	Female	55 (29.9)
Ethnicity	Brahmin	24 (13)
	Chhetri	36 (19.6)
	Janajati	87 (47.3)
	Dalit	13 (7.1)
	Others	24 (13)
Religion	Hindu	137 (74.5)
	Buddhist	32 (17.4)
	Muslim	5 (2.7)
	Christianity	10 (5.4)
Marital status	Married	129 (70.1)
	Unmarried	55 (29.9)
Education	Illiterate	18 (9.8)
	Primary	51 (27.7)
	Secondary	58 (31.5)
	Campus	57 (31)

**Table 2:** Respondents’ knowledge of dermatophytosis.

SN	Questions	Yes (%)	No (%)
1	Does it spread from person to person?	80.4	19.6
2	Is it seasonal?	45.7	54.3
3	Is sweating related to this infection?	72.8	27.2
4	Does sharing of clothes and objects spread it?	77.2	22.8
5	Does collective soaking and washing of clothes of all family members increase the risk of getting infected?	73.9	26.1
6	Knowledge of possible sites of dermatophytosis	48.4	51.6
7	Can certain occupations increase the risk of dermatophytosis?	36.4	63.6
8	Can dermatophytosis affect hair?	58.2	41.8

**Table 3:** Dermatophytosis-related hygiene practices.

SN	Hygiene practices	Yes (%)	No (%)
1	Do you bath daily?	41.8	58.2
2	Do you wash clothes after every use?	59.2	40.8
3	Do you wear ironed clothes	26.6	73.4
4	Do you wash your feet after coming from outside?	86.4	13.6
5	Do you have a bath after getting drenched in sweat?	83.7	16.3
6	Do you share clothes/towels?	20.7	79.3
7	Do you share your hairbrush/comb?	16.8	83.2
8	Are you following any prescribed treatments for fungal skin infections?	48.4	51.6
9	Clothes and undergarments drying practice	Percentage (%)	
	a. In the sun outside	92.9	
	b. Inside rooms on stand	7.1	
10	Nails trimming frequency		
	a. Once a week	77.7	
	b. Once every 15 days	3.8	
	c. Once a month	18.5	
11	Washing towels frequency		
	a. Monthly	4.9	
	b. Weekly	85.9	
	c. Every 15 days	9.2	

**Table 4:** Comparison of socio-demographic variables and knowledge on dermatophytosis.

Variables	Dermatophytosis-related knowledge status		*p* value
	Good knowledge-*N* (%)	Poor knowledge-*N* (%)	
Age category			
<20 Years	4 (2.2)	2 (1.1)	0.517
20–40 Years	80 (43.5)	46 (25)	
40–60 Years	22 (12)	21 (11.4)	
>60 Years	6 (3.3)	3 (1.6)	
Sex			
Male	89 (48.4)	40 (21.7)	0.001*
Female	23 (12.5)	32 (17.4)	
Ethnicity			
Brahmin	16 (8.7)	8 (4.3)	0.854
Chhetri	23 (12.5)	13 (7.1)	
Janajati	50 (27.2)	37 (20.1)	
Dalit	9 (4.9)	4 (2.2)	
Others	14 (7.6)	10 (5.4)	
Religion			
Hindu	84 (45.7)	53 (28.8)	0.013*
Buddhist	14 (7.6)	18 (9.8)	
Muslim	5 (2.7)	0 (0.0)	
Christianity	9 (4.9)	1 (0.5)	
Education			
Illiterate	10 (5.4)	8 (4.3)	0.206
Primary	30 (16.3)	21 (11.4)	

Bold values are statistically significant. *p-value <0.05.

**Table 5:** Comparison of socio-demographic variables and infection status.

Variables	Infection status		B coefficient	95%CI	*p* value
	Yes (%)	No (%)			
Age category					
<20 Years	0 (0)	6 (3.3)	0.547	0.799–3.742	0.165
20–40 Years	41 (22.4)	85 (46.2)			
40–60 Years	27 (14.6)	16 (8.7)			
>60 Years	1 (0.5)	8 (4.3)			
Gender					
Male	25 (19.4)	104 (80.6)	2.954	7.065–52.134	0.001*
Female	44 (80)	11 (20)			
Ethnicity					
Brahmin	10 (5.4)	14 (7.6)	0.091	0.737–1.629	0.653
Chhetri	11 (6)	25 (13.6)			
Janajati	33 (17.9)	54 (29.3)			
Dalit	4 (2.2)	9 (4.9)			
Others	11 (6.0)	13 (7.1)			
Religion					
Hindu	52 (28.3)	85 (46.2)	−0.266	0.401–1.465	0.421
Buddhist	13 (7.1)	19 (10.3)			
Muslim	1 (0.5)	4 (2.2)			
Christianity	3 (1.6)	7 (3.8)			
Education					
Illiterate	7 (3.8)	11 (6)			0.635
Primary	34 (10.9)	17 (16.8)			
Secondary	39 (12)	19 (19.6)			
Campus	46 (10.9)	11 (20.1)			
Smoking status					
Smoker	25 (13.6)	71 (38.6)	−0.252	0.321–1.882	0.577
Non-smoker	44 (23.9)	44 (23.9)			
Knowledge category					
Good knowledge	27 (14.7)	85 (46.2)	−1.286	0.115–0.662	0.004*
Poor knowledge	42 (22.8)	30 (16.3)			
Practice category					
Good hygiene	42 (22.8)	84 (45.7)	−1.171	0.129–0.746	0.009*
Poor hygiene	27 (14.7)	31 (16.8)			

Bold values are statistically significant. *p-value <0.05.

### Knowledge regarding dermatophytosis

Assessment of prisoners’ knowledge regarding the spread of fungal infection from person to person showed that the majority (80.4%) of participants responded correctly. The study also revealed that 72.8% of prisoners reported correctly that sweating is related to infection with dermatophytosis. One-third of respondents had good knowledge regarding the spread of dermatophytosis due to collectively sharing clothes and washing. Around half of the respondents had good knowledge of the seasonal nature and possible sites of dermatophytosis infection. 63.6% of respondents did not have correct knowledge regarding occupation-related dermatophyte infection.

### Dermatophytosis-related personal hygiene practices

About 41.8% of respondents said they bathed daily, 59.2% reported washing clothes after every use, and 26.6% stated that they wore ironed clothes. Most of them habitually washed their feet after coming from outside (86.4%) and bathed after getting drenched in sweat (83.7%). One-fifth of respondents shared their clothes/towels or comb. Most respondents (77.7%) reported trimming their nails weekly. A considerable proportion of the respondents (92.9%) revealed drying clothes in the sun outside. Only 7.1% reported drying clothes inside rooms. Most of them (85.9%) reported washing towels weekly.

### Comparison of sociodemographic variables and knowledge category

The study explored the relationship between demographic factors and knowledge levels about skin diseases, revealing several significant associations. Notably, the gender exhibited a significant association (*p* = 0.001), with females showing higher susceptibility than males. Furthermore, religion was significantly associated with skin disease status (*p* = 0.013), indicating that individuals belonging to the Hindu faith had a higher prevalence of skin diseases. Smoking status also showed a significant association (*p* = 0.022), with smokers exhibiting a higher risk. However, no significant associations were found between knowledge status and ethnicity, marital status, education and comorbidities category, with individuals aged 20–40 showing higher susceptibility. Gender also exhibited a notable association (*p* < 0.001), with females being more prone to skin diseases (B coefficient = 2.954, 95% CI [7.065, 52.134], *p* = 0.001). Moreover, the knowledge category demonstrated significance (*p* = 0.004), indicating that individuals with poor knowledge were at a higher risk (B coefficient = −1.286, 95% CI [0.115, 0.662], *p* = 0.004). Practice category also played a role (*p* = 0.009), with poor hygiene practices correlating with increased susceptibility (B coefficient = −1.171, 95% CI [0.129, 0.746], *p* = 0.009). However, no significant associations were found between skin disease status, ethnicity, and religion.

## Discussion

The study’s findings provide valuable insights into the knowledge, infection status and hygiene practice on dermatophytosis among prisoners. The mean age of respondents was 35, close to that reported by Okareh et al. (10). Younger individuals (20–40 years) demonstrated higher susceptibility to skin diseases, which could be attributed to lifestyle factors and increased exposure. The findings are from the study conducted by SK et al. and Paudel et al. (8, 11). We also report that our study had more female cases (80%) of dermatophytosis than male cases (20%). It is noted in similar studies conducted by SK et al. and Pires et al. (2, 8) but not in other studies (11–14). Individuals belonging to the Hindu faith showed a higher prevalence of skin diseases, highlighting the potential influence of cultural and religious practices on disease susceptibility. Smoking status was also found to be a significant factor, with smokers being at a higher risk, which could be attributed to the immunosuppressive effects of smoking.

Overall, the study revealed that a significant proportion of respondents (60.9%) had good knowledge regarding the spread and prevention of dermatophytosis, with the majority correctly identifying the mode of transmission and the importance of personal hygiene practices. This could be explained by 62.5% of the respondents having education up to a secondary level or more. 80% of participants knew about the spread of fungal infection from person to person, and 95% of respondents believed that treatment is necessary for dermatophytosis. It is coherent with Yamu et al. but not consistent with SK et al. 45% had good knowledge of the seasonal nature of dermatophytosis, similar to SK et al., but differs from Yamu et al. (8, 15). However, there were also areas where knowledge gaps were evident, particularly regarding occupation-related dermatophyte infections.

The study also highlighted the importance of hygiene practices in preventing dermatophytosis. Practices such as daily bathing, washing clothes after every use, and avoiding sharing clothes/towels were associated with better knowledge levels and lower susceptibility to skin diseases. 68.5% of respondents had good hygiene practices, which might indicate satisfactory hygiene services in the prison settings (16–20). The percentage of respondents bathing daily is less than that of Yamu et al. and SK et al. It can be explained by the winter season at the time of data collection. 92.9% of respondents dried clothes in the sun, similar to Yamu et al. and SK et al. (8, 15). However, a significant proportion of respondents reported suboptimal hygiene practices, indicating the need for targeted interventions to promote better hygiene practices among this population (16–20).

### Limitations

Due to the cross-sectional study design, causal inference regarding any observed association between dependent and independent variables cannot be established. The findings are also limited in generalizability to the broader population because the study was conducted on prisoners in a specific setting. The infection status was not assessed on basis of particular diagnosis of individual disease.

## Conclusion

In conclusion, this study provides valuable information about Nepal’s Central Prison prisoners’ dermatophytosis-related knowledge and hygiene habits. It emphasizes how a person’s age, gender, degree of awareness, and cleanliness habits significantly impact their susceptibility to dermatophytosis. Specifically, males and individuals aged 20–40 were identified as more prone to skin diseases. Moreover, poor knowledge and hygiene practices were also linked to increased dermatophytosis risk. Considering these facts highlights the critical need for comprehensive strategies for preventing and managing dermatophytosis.

### Recommendations

Based on our research findings, we suggest special educational programs on dermatophytosis prevention and treatment for prisoners in Central Prison. These programs should address myths, promote hygienic practices, and encourage positive attitudes toward dermatophytosis management. In addition, routine screenings and timely medical care should be offered to prevent the disease from spreading inside the prison setting.

## Data availability statement

The raw data supporting the conclusions of this article will be made available by the authors, without undue reservation.

## Ethics statement

The studies involving humans were approved by the Nepal Health Research Council (NHRC)’s ethical review board. The studies were conducted in accordance with the local legislation and institutional requirements. Written informed consent for participation in this study was provided by the participants’ legal guardians/next of kin.

## Author contributions

BD: Conceptualization, Data curation, Formal analysis, Investigation, Methodology, Project administration, Resources, Validation, Visualization, Writing – original draft, Writing – review & editing. BS: Data curation, Investigation, Project administration, Validation, Writing – original draft, Writing – review & editing. SS: Formal analysis, Investigation, Project administration, Software, Validation, Writing – original draft, Writing – review & editing. SR: Data curation, Formal analysis, Methodology, Software, Writing – original draft, Writing – review & editing. BK: Data curation, Funding acquisition, Methodology, Project administration, Supervision, Writing – original draft, Writing – review & editing. PP: Formal analysis, Investigation, Project administration, Supervision, Writing – original draft, Writing – review & editing. NB: Writing – review & editing, Formal analysis, Investigation, Project administration, Software, Writing – original draft. RM: Investigation, Project administration, Supervision, Writing – original draft, Writing – review & editing. NT: Data curation, Funding acquisition, Methodology, Resources, Writing – original draft, Writing – review & editing. BZ: Data curation, Funding acquisition, Project administration, Resources, Writing – original draft, Writing – review & editing. RS: Formal analysis, Methodology, Writing – original draft, Writing – review & editing. CL: Investigation, Methodology, Writing – original draft, Writing – review & editing. DB-A: Conceptualization, Formal analysis, Methodology, Supervision, Writing – original draft, Writing – review & editing. AR-M: Data curation, Investigation, Methodology, Resources, Supervision, Writing – original draft, Writing – review & editing.
